# Unveiling Ureaplasma: A Case Report of a Rare Culprit in Pyelonephritis

**DOI:** 10.7759/cureus.54958

**Published:** 2024-02-26

**Authors:** Manish Shrestha, Devi Parvathy Jyothi Ramachandran Nair, Shefali Amin, Arpan Pokhrel, Salina Munankami

**Affiliations:** 1 Internal Medicine, Tower Health Medical Group, Reading, USA; 2 General Medicine, Kathmandu Medical College, Kathmandu, NPL

**Keywords:** antimicrobial resistance, urinary tract infection, pcr test, complicated pyelonephritis, ureaplasma urealyticum

## Abstract

*Ureaplasma* species, typically considered commensal organisms of the human urogenital tract, have been implicated in various urinary tract infections (UTIs), including the rare and challenging presentation of pyelonephritis. This case report describes a unique instance of pyelonephritis induced by *Ureaplasma*, characterized by a negative routine urine culture and a lack of response to empirical antibiotic treatment, highlighting the complexities associated with diagnosing and managing infections caused by atypical pathogens. A 50-year-old female presented to the emergency department with symptoms suggestive of UTI, including fever, vomiting, and dysuria. However, initial urine analysis was notable for pyuria while routine bacterial culture returned negative results, creating a diagnostic dilemma. Empirical treatment with third-generation cephalosporin was initiated. However, the patient's condition failed to improve, raising concerns about antibiotic resistance or atypical pathogens. Subsequent molecular diagnostics, precisely polymerase chain reaction (PCR), identified *Ureaplasma urealyticum* as the causative agent. This prompted a change in the treatment regimen to doxycycline, to which the patient showed significant clinical improvement. Physicians should be aware of *Ureaplasma* as a potential cause of pyelonephritis, especially in cases of culture-negative UTIs and when patients do not respond to standard empirical treatment. This case emphasizes the importance of considering atypical pathogens in differential diagnosis and the role of molecular diagnostic techniques in guiding appropriate management.

## Introduction

*Ureaplasma urealyticum*, a member of the family *Mycoplasmataceae*, is a small, wall-deficient bacterium that is a common inhabitant of the human urogenital tract [1]. Although often considered a commensal organism, *Ureaplasma* has been implicated in various clinical conditions, ranging from asymptomatic colonization to severe infections such as non-gonococcal urethritis and pyelonephritis [1]. Pyelonephritis, an infection of the upper urinary tract, typically presents with symptoms of fever, flank pain, and dysuria and is most commonly caused by *Escherichia coli* with the standard approach to diagnosis being urine culture, which guides targeted antimicrobial therapy [2].

However, the diagnosis and management of pyelonephritis caused by atypical pathogens such as *Ureaplasma* are challenging as the infection has atypical presentation, such as a negative urine culture or resistance to empiric antibiotic therapy, which is designed to cover common uropathogens [3]. The case of *Ureaplasma*-induced pyelonephritis with negative culture results and non-responsiveness to empiric treatment is unique and noteworthy as it underscores the diagnostic limitations of standard urine cultures in detecting *Ureaplasma* species, which require specific culture media and conditions for growth [1,3]. Moreover, it highlights the organism's potential for pathogenicity in the urinary tract, challenging the prevailing notion of *Ureaplasma* as a harmless commensal [1,3]. Thus, the case illustrates the importance of considering atypical pathogens in patients with pyelonephritis who do not respond to standard empiric therapy.

## Case presentation

A 50-year-old sexually active woman with a history of recurrent urinary tract infection (UTI) and bacterial vaginosis presented to the hospital with fever, chills, nausea, and vomiting for three days. These symptoms were associated with burning urination and increased urinary frequency. She denied having hematuria and abdominal pain but experienced mild discomfort in the suprapubic area. Initially seen as an outpatient, she was prescribed 500 mg of ciprofloxacin twice daily for her symptoms. She took a single dose before presenting to the emergency department due to worsening symptoms. She has been taking nitrofurantoin 100 mg as postcoital prevention for her recurrent urinary tract infections for a few years.

Upon presentation, her temperature was 100.7°F, heart rate was 82 beats per minute, respiratory rate was 18 breaths per minute, and blood pressure was 111/70 mmHg, with an oxygen saturation of 98% on room air. She was awake, alert, and oriented but appeared fatigued. The examination revealed left costovertebral angle tenderness and mild lower abdominal tenderness with normal bowel sounds. She did not exhibit rebound tenderness. Laboratory analysis (Table 1) showed an unremarkable basic metabolic panel, while the complete blood count revealed elevated white blood cells at 15.6 x 10^9^/L. Urinalysis revealed cloudy urine with moderate blood. Microscopic urine assessment found occasional mucus and six white blood cells per high-power field, and it was negative for nitrites, leukocyte esterase, and bacteria.

**Table 1:** Laboratory Analysis

Laboratory Test	Value	Reference Range
Sodium (mmol/L)	137	136-145
Potassium (mmol/L)	3.4	3.5-5.1
Chloride (mmol/L)	107	98-107
Blood Urea Nitrogen (mg/dL)	9	7-25
Creatinine (mg/dL)	0.61	0.6-1.3
Glucose (mg/dL)	107	70-99
Calcium (mg/dL)	8.3	8.6-10.3
Albumin (g/dL)	3.3	3.5-5.7
Total protein (g/dL)	6	6.4-8.9
Aspartate aminotransferase (IU/L)	65	13-39
Alanine aminotransferase (IU/L)	54	7-52
Alkaline phosphatase (IU/L)	62	34-104
White blood cells (x 10^3^/uL)	15.6	4.8-10.8
Red blood cells (x 10^6^/uL)	3.61	4.5-6.1
Hemoglobin (g/dL)	10.2	12-16
Platelets (x 10^3^/uL)	125	130-400

**Figure 1:**
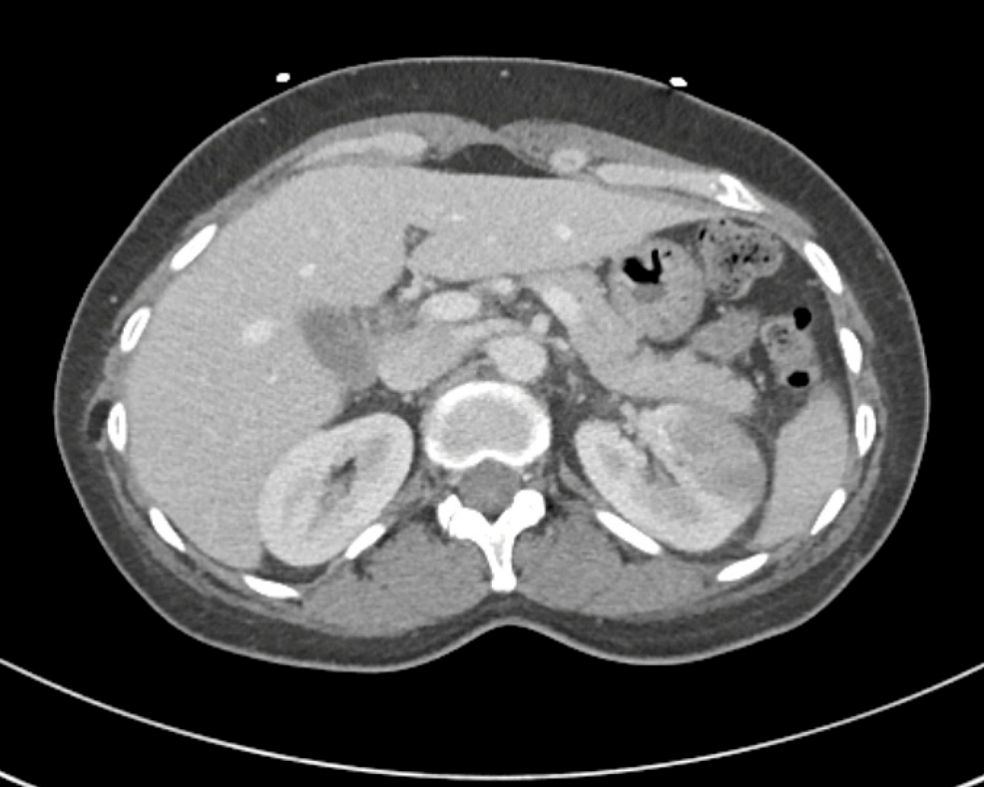
CT scan of abdomen and pelvis revealing areas of low-attenuation within the anterior lateral and posterior medial mid-left kidney suggestive of acute pyelonephritis

**Figure 2:**
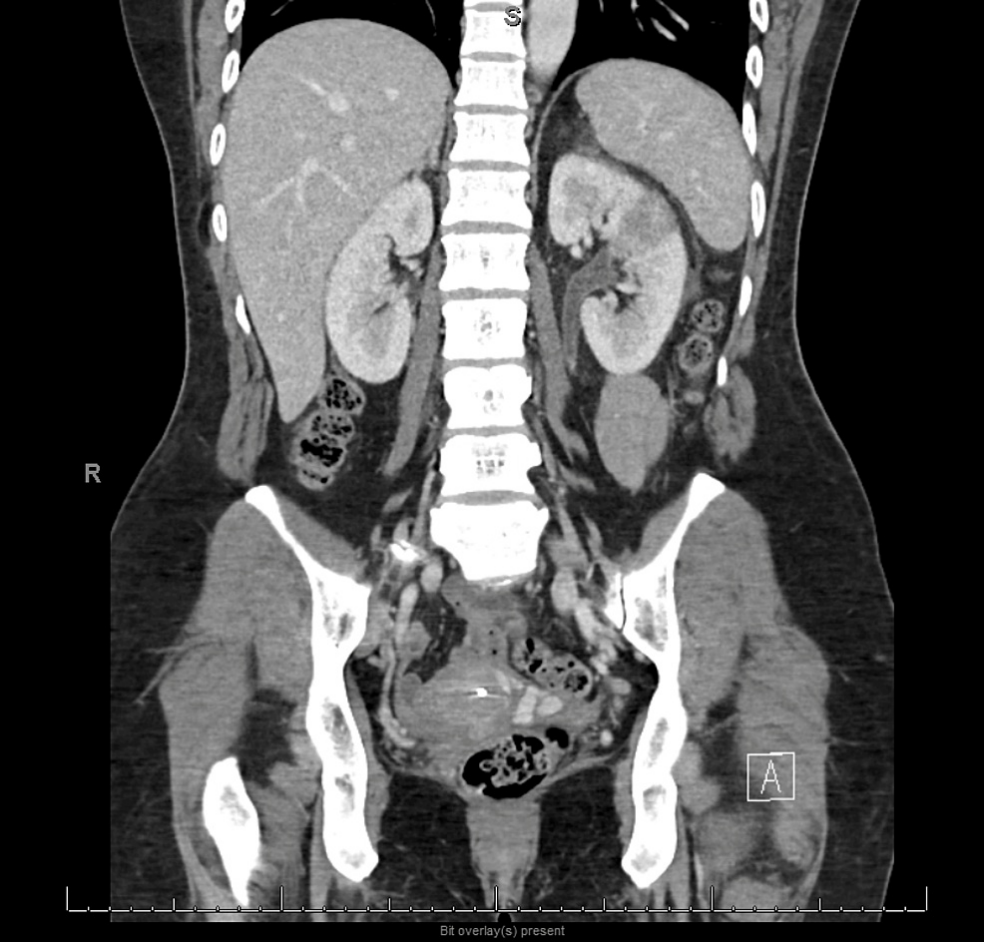
CT scan of abdomen and pelvis showing mild left ureteral wall thickening secondary to ureteritis

Computed tomography of the abdomen and pelvis with contrast showed findings consistent with left-sided pyelonephritis (Figure 1) and associated urethritis (Figure 2), as well as a suggestion of chronic pelvic venous congestion syndrome. The patient was admitted with a primary working diagnosis of sepsis secondary to left-sided pyelonephritis and was treated with intravenous fluids and empirical antibiotic coverage with IV ceftriaxone. Urine culture results showed no growth. She was discharged on oral cefpodoxime to complete a 10-day course.

One week later, she presented to the outpatient internal medicine office with persistent symptoms of dysuria and urinary frequency, even after she had completed the prescribed course of antibiotics. The examination also revealed mild left costovertebral angle tenderness. There was a growing suspicion of atypical organisms like *Ureaplasma* and *Mycoplasma*, and tests were conducted accordingly. Polymerase chain reaction (PCR) testing on the urine sample returned positive for *U. urealyticum*. She was started on a 10-day course of oral doxycycline. Upon follow-up after a week, the patient had successfully recovered from the treatment. Considering her recurrent UTI symptoms, we suspect that untreated *Ureaplasma* may have been the cause of her longstanding urethritis and might have contributed to the reflux pyelonephritis.

## Discussion

*Ureaplasma* species, particularly *U. urealyticum* and *U. parvum*, are common inhabitants of the human urogenital tract [1]. While often commensal, these microorganisms have been implicated in various UTIs, including urethritis, cystitis, and, more rarely, pyelonephritis [1,2]. The precise prevalence of *Ureaplasma*-induced pyelonephritis remains difficult to ascertain due to diagnostic challenges [4]. *Ureaplasma* species are frequently isolated from the urogenital tract, with varying colonization rates reported in sexually active adults, with up to 80% of healthy females having *Ureaplasma* species in their cervical or vaginal secretions [5]. Despite their prevalence, the incidence of these bacteria as causative agents in pyelonephritis cases is not well-documented, partly because of limitations in routine bacterial culture methods [5].

*Ureaplasma* species possess several virulence factors that facilitate colonization of the urogenital tract and evasion of host immune responses [6]. The organisms' ability to adhere to uroepithelial cells and produce urease contributes to urinary tract colonization and infection by increasing urinary pH, promoting the formation of struvite stones, and creating a favorable environment for bacterial proliferation [6,7]. However, the mechanisms by which *Ureaplasma* species ascend to the upper urinary tract and induce pyelonephritis are not fully understood and warrant further investigation. Diagnosing *Ureaplasma*-induced pyelonephritis is complicated by the organisms' fastidious growth requirements, which are not met by standard UTI culture protocols [8]. Molecular methods such as PCR offer greater sensitivity and specificity but are only routinely available in some clinical settings ([8,9]. The development of rapid, accurate, and cost-effective diagnostic tests remains a critical need in managing Ureaplasma-associated UTIs. Treatment of Ureaplasma-induced pyelonephritis typically involves antibiotics such as tetracyclines and macrolides, to which these organisms are generally susceptible ([10]. However, emerging resistance to these antibiotics poses a significant challenge, and the lack of standardized treatment guidelines for *Ureaplasma* infections complicates the management of these cases, highlighting the need for further research on effective therapeutic strategies ([10,11].

The clinical outcomes of *Ureaplasma*-induced pyelonephritis vary, ranging from complete resolution with appropriate antibiotic therapy, like in this case, to chronic infection and renal scarring in cases of delayed diagnosis or treatment failure [12].

## Conclusions

*Ureaplasma*-induced pyelonephritis represents a complex and underrecognized clinical entity. Current challenges include improved diagnostic methods, effective treatment regimens, and a deeper understanding of the pathogenesis of Ureaplasma infections. Addressing these issues through continued research and clinical vigilance is essential for optimizing patient care and outcomes in *Ureaplasma*-associated UTIs.
